# A cross-sectional survey of parental attitudes towards Human papillomavirus vaccination exclusion categories in Brazil

**DOI:** 10.1186/s12914-019-0195-5

**Published:** 2019-02-28

**Authors:** Mariana V. Gattegno, Maria A. F. Vertamatti, Robert A. Bednarczyk, Dabney P. Evans

**Affiliations:** 1 Emory University, Rollins School of Public Health, 1518 Clifton Rd NE, Mailstop 1518-002-7BB, Atlanta, GA 30322 USA; 2 Faculdade de Medicina do ABC, Av. Príncipe de Gales, 821, Santo André, SP 09060-650 Brazil

**Keywords:** Brazil, Universal health coverage, Human papillomavirus, Vaccination, Adolescent health, Parental perceptions, Human rights

## Abstract

**Background:**

In 1988, Brazil established a constitutional right to health and universal access to health care for all Brazilians through the creation of the Unified Health System (SUS). As part of its efforts to fulfill this right, the quadrivalent Human papillomavirus (HPV) vaccine was introduced into the national immunization program in 2014. The non-discriminatory provision of healthcare goods, facilities, and services is a fundamental part of the right to health. Yet HPV vaccination was limited to females aged 9–13, despite the universal nature of SUS and scientific support for the vaccination of males and older females. The purpose of this cross-sectional study was to describe parental attitudes regarding age- and gender-based HPV vaccination exclusions, as well as parental knowledge of HPV and the HPV vaccine.

**Methods:**

Data were gathered from parents with children aged 9–17 in a health post located in the municipality of Mauá (São Paulo, Brazil) through interviewer-administered questionnaires. We analyzed attitudes regarding HPV vaccination and its eligibility guidelines by comparing parents of HPV vaccine eligible and ineligible children.

**Results:**

In this low-income population, the majority of the 219 parents surveyed supported the inclusion of males and females over 13 into the HPV vaccination program; support for the non-discriminatory provision of the HPV vaccine was high among parents – especially if financially accessible. Additionally, there were high levels of knowledge and positive parental attitudes regarding HPV vaccination safety and efficacy among both parent groups suggesting information accessibility – a key component of the right to health and informed decision-making.

**Conclusions:**

Support for the expansion of HPV vaccination for excluded populations exists, and is not based on current eligibility, or differential knowledge and attitudes about the vaccine. Moving forward, careful consideration of gender- based eligibility for vaccination, informed decision-making, and the importance of community participation in health policy development and implementation may be gleaned from the case of Brazil and beyond.

## Background

In 1948, both the World Health Organization (WHO) Constitution and the Universal Declaration of Human Rights described health as a fundamental human right [1, 2]. Article 12 of the International Covenant on Economic Social and Cultural Rights (ICESCR) (1966) further enumerated the right [2]. Participation, financial accessibility, information accessibility, and non-discrimination are among the key principles that states must uphold as a matter of their right to health obligations [3]. Substantively, the right to health includes protection of the wellbeing of children, efforts to adequately immunize against infectious diseases, specific health issues such as sexually transmitted diseases, and the creation of conditions to ensure equal and universal access to health care [4, 5]. Each of these is relevant to the topic of vaccination, particularly vaccination against Human papillomavirus (HPV) to prevent cervical cancer.

In Brazil, cervical cancer is the second most common cancer among women. Incidence and mortality of cervical cancer in Brazil are the highest in the Americas; the mortality rate is 8.3 deaths per 100,000 women [6, 7]. Infection with HPV types − 16 and − 18, which are responsible for approximately 70% of cervical cancer cases, can be prevented through vaccination [8]. The targeted HPV vaccination of female adolescents serves as the lens through which this paper identifies potentially discriminatory processes in the implementation of the right to health by the Brazilian public health system. We pay specific attention to age- and gender-based discrimination, financial accessibility, information accessibility, and community participation in HPV policy development and programming through the Brazilian public health system.

The *Sistema Único de Saúde* (Unified Health System; SUS) was established as a part of the Brazilian constitutional guarantee of the right to health [9]. SUS provides health coverage to about 75% of the population, granting free access to medication, health technology, health checks, immunizations, and treatments [10]. SUS maintains community-based health posts that are all-inclusive medical units, which include family health and vaccination [10]. SUS also sponsors the *Programa Nacional de Imunização (*National Immunization Program; PNI) that is responsible for establishing the national vaccine schedule; the current national schedule includes 26 vaccines, including the quadrivalent HPV vaccine [11].

In 2014, Brazil joined 70 other countries by introducing the quadrivalent HPV vaccine into the PNI. Free of cost to the individual, the national school-based vaccination program was targeted at females aged 9–13 [12, 13]. The campaign has been incredibly successful with some states nearing 100% coverage of the eligible population for the first dose [11]. Despite the universal nature of SUS – and the vaccine’s proven efficacy in males and females up to age 26 – this program was limited to early-adolescent females, ignoring the Constitution’s ‘right to health’ guarantee [13]. Moreover, the non-discriminatory provision of healthcare goods, facilities, and services is a fundamental part of the right to health. Since males are the primary carriers of HPV, excluding them from vaccination via SUS represents, at best, a missed opportunity to improve protection of females and also prevent anogenital warts and penile, anal, and oropharyngeal cancers in males [13–15]. At worst, the exclusion of males represents a violation of the right to health through the discriminatory omission of a population vulnerable to HPV infection. Currently, eleven countries have introduced HPV vaccination programs for both males and females [13]. WHO suggests that decisions on gender-neutral vaccine recommendations should be based on a country-level analysis and decision [13].

The current vaccination policy does not support the universal and equitable access to health care espoused by the SUS system, the Brazilian constitution, and international human rights law [2, 3, 9]. The policy also ignores the state obligation for participation by affected populations, including in setting health program priorities and strategies [3]. Moreover, the exclusion of certain populations is in contrast to the WHO recommendation to administer the quadrivalent vaccine to females and males aged 9–13 years according to a 2-dose schedule and according to a 3-dose schedule for females and males aged 14 years or older (up to age 26) [13]. The purpose of the study was to describe parental attitudes regarding age- and gender-based HPV vaccination exclusions, as well as parental knowledge of HPV and the HPV vaccine.

## Methods

Between June and August 2015, we conducted an interviewer administered cross-sectional survey with 219 participants to understand parental knowledge and attitudes regarding HPV vaccination. Survey topics included: attitudes regarding HPV vaccination for excluded populations, knowledge of HPV, current HPV vaccination, and HPV vaccination eligibility. Parents and guardians (hereafter, “parents”) of children aged 9–17 attending health posts in Mauá, Brazil were eligible to participate. The lower age range was selected following SUS guidelines recommending HPV vaccination for females aged 9–13 years, while the upper age range was selected since parents are still legally responsible for their children until age 18.

The project site was Unidade de Atencão Básica de Saúde (primary health post; UBS) Kennedy, located in Mauá, a municipality in the state of São Paulo on Brazil’s east coast [16]. UBS Kennedy operates with four Family Health teams, which see approximately 120 patients per day from various backgrounds and socioeconomic statuses. UBS Kennedy was selected by local partners due its size and location; it is one of the larger health post in the municipality of Mauá, and is centrally located in reference to the surrounding communities.

Following an extensive review of relevant studies, the survey instrument was comprised of original questions and adapted items from existing instruments that measured attitudes and parental decision making related to the HPV vaccine [17–19]. The instrument included five major sections: demographics, HPV and HPV vaccine knowledge, perceived vaccine safety/efficacy, and excluded populations. The survey was translated into Portuguese by a native speaker and back translated to English; we pilot tested the survey and made adjustments where needed. The instrument is available upon request.

Of approximately 100 patients who visited the clinic each day, approximately 8 to 10 were recruited for participation. Each individual that entered the clinic was approached for participation by the researcher, who verified eligibility and provided an overview of the study. Due to low literacy levels, consent to participate was obtained from each participant by verbally reviewing the consent form, providing all pertinent information to the subject and allowing the subject the opportunity to ask questions. Participants were required to sign (or print) their names at the bottom of the consent form before beginning the survey, and were provided with a copy of the consent form if requested. The face-to-face survey was administered in Portuguese; the interviewer read each survey item aloud and recorded the participant’s responses on the survey instrument. Sample size was determined based on a 95% confidence level and a 5% margin of error.

Consent documents and questionnaires were filed separately from one another in a locked filing folder to ensure confidentiality. Survey data were entered into Excel and stored in a password-protected folder on a password-protected laptop. The Excel file was uploaded to SAS version 9.4 (The SAS Institute, Cary NC) for data cleaning and analysis. Completed paper surveys were filed at the Brazilian partner organization (FMABC) in accordance with the requirements of the Mauá Ethics Review Board.

For analysis, parents were divided into two categories: “HPV vaccine eligible” and “HPV vaccine ineligible.” “HPV vaccine eligible” parents had one or more female children aged 9–15 eligible for HPV vaccination during the course of the study. While the 2016 HPV vaccination policy only included females aged 9 and 13, females who were in this age range in 2014 (but no longer eligible for vaccination) could have been up to age 15 at the time of the study; therefore their parents were included in the “HPV vaccine eligible” category. All other parents were considered “HPV vaccine ineligible.”

Descriptive statistics were computed and stratified by HPV parent eligibility. We conducted bivariate analyses with chi-square tests (or Fisher’s Exact tests, where appropriate). Significance was assessed at α = 0.05 level. Willingness to vaccinate all individuals age 9–26 was examined by parent’s demographic, knowledge, and attitude variables.

The project was reviewed and approved by the Emory University Institutional Review Board (Atlanta, GA, USA) and the Municipality of Mauá (São Paulo, SP, Brazil).

## Results

### Demographics

Two-hundred nineteen (*N* = 219) parental surveys were included in the data analysis, with responses relating to 432 children. Most (87.2%) parents were female; ages ranged from 25 to 64 years. Approximately 30% of participants were either unemployed or making less than the Brazilian minimum wage (US$203 monthly); median income was US$258 (IQR: US$116 to $413). Most parents (62%) indicated they were responsible for health-related decisions regarding the children in the house (Table 1).

**Table 1:** Demographic characteristics of parents with children between the ages of 9 and 17 in São Paulo, Brazil, 2015, stratified by child eligibility for the HPV vaccine through SUS

Characteristic	Level	Overall (*N* = 219)		HPV vaccine eligible (*N* = 115)		HPV vaccine ineligible (*N* = 104)	
		No.	%	No.	%	No.	%
Gender	Female	191	87.2	97	84.4	94	90.4
	Male	28	12.8	18	15.6	10	9.6
Age	25–29	32	14.6	22	19.1	10	9.6
	30–34	43	19.6	24	20.9	19	18.3
	35–39	63	28.8	35	30.4	28	26.9
	40–44	36	16.4	14	12.2	22	21.1
	45–49	27	12.3	10	8.7	17	16.4
	50 or older	18	8.2	10	8.7	8	7.7
Marital status^a^	Single	74	33.9	40	35.1	34	32.7
	Married	144	66.1	74	64.9	70	67.3
Responsible for health-related decisions	Self	136	62.1	66	57.4	70	67.3
	Spouse	10	4.6	5	4.3	5	4.8
	Together	73	33.3	44	38.3	29	27.9
Number of children	1–2	109	49.8	56	48.7	53	50.9
	3–4	96	43.8	51	44.3	45	43.3
	5 or more	14	6.4	8	7.0	6	5.8
Age of children^b^	9–15	251	43.4	163	52.4	88	32.8
	16–20	101	17.4	33	10.6	68	25.4
	21 or older	80	13.8	32	10.3	48	17.9
Gender of children^c^	Female	298	51.5	205	65.9	175	65.3
	Male	281	48.5	106	34.1	93	34.7
		No.	USD	No.	USD	No.	USD
Monthly income	Median	184	$258.20	100	$236.60	84	$271.10

^a^*n* = 218

^b, c^*n* = 579

While the study was focused on parents of children aged 9–17, we collected demographic information about the age and sex of all children parents were responsible for. Children’s ages ranged from newborn (age calculated in months) to 21 years of age or older, with the majority aged 9–15 years (*n* = 251 children) and 51.5% being female. There were no significant demographic differences between the two groups thus allowing for cross group comparison of the attitudinal and knowledge variables (*p* > 0.05) (Table 1).

### Attitudes regarding excluded populations

Overall, parents had positive attitudes regarding the HPV vaccine and its provision to excluded populations (males and older females). Ninety-four percent (94%) of parents said they would pay to have their children vaccinated if the child did not qualify for vaccination through SUS; however, parents stipulated that they would do so only if they could afford the vaccine. Approximately 89% of parents also said that they would have all their children - regardless of gender and age - vaccinated against HPV if the cost of the vaccine was covered through SUS. Over 86% of parents expressed that they had no hesitation in regards to the HPV vaccine. Across the two parent groups there were no statistically significant differences about the provision of the HPV vaccine to currently excluded populations (*p* > 0.05) (Table 2).

**Table 2:** Attitudes of parents with children between the ages of 9 and 17 regarding the HPV vaccine and its provision to currently excluded populations

	Overall N (%)	HPV vaccine eligible N (%)	HPV vaccine ineligible N (%)	FET *p*-value
Would you pay to have your children vaccinated against HPV if they were not eligible for free vaccination? (*N* = 216)				0.5679
Yes	203 (94.0)	108 (95.6)	95 (92.2)	
No	10 (4.6)	4 (3.5)	6 (5.8)	
Not Sure	3 (1.4)	1 (0.9)	2 (1.9)	
If the vaccine was offered at no cost to all individuals between the ages of 9 and 26, would you have *all* of your children vaccinated against HPV? (*N* = 215)				0.2495
Yes, all my children	191 (88.8)	97 (85.8)	94 (92.2)	
Yes, but only the females	15 (7.0)	11 (9.7)	4 (3.9)	
Yes, but only the males	1 (0.5)	1 (0.9)	0	
No	8 (3.7)	4 (3.5)	4 (3.9)	
Do you have any hesitations in regards to the HPV vaccine? (N = 216)				0.0925
Yes	23 (10.6)	17 (15.0)	6 (5.8)	
No	187 (86.6)	93 (82.3)	94 (91.3)	
Not Sure	6 (2.8)	3 (2.7)	3 (2.9)	

### Attitudes regarding vaccination

Parents had overall positive attitudes regarding vaccination. Both parent groups agreed that it is important to vaccinate adolescents in general, as well as vaccinate against HPV infection (over 96% agreement for both questions). Additionally, both groups agreed that HPV vaccination is beneficial for females aged 9–13 (approximately 94% agreement), as well as beneficial for females over 13 years (approximately 83% agreement). HPV vaccine eligible and HPV vaccine ineligible parents differed slightly in their beliefs regarding vaccine efficacy in preventing cervical cancer, vaccine safety, men being vaccinated against HPV, and vaccine efficacy for men. However, none of these attitudinal differences were statistically significant across parent groups (*p* > 0.05) (Table 3). It should be noted that some of the questions included in this section of the survey contain factual statements; however, the attitudes around them can impact parental vaccination decisions and were therefore included as attitudes rather than knowledge.

**Table 3:** Attitudes about vaccination and the HPV vaccine among parents with children between the ages of 9 and 17 in São Paulo, Brazil, 2015

	HPV vaccine eligible N (%)			HPV vaccine ineligible N (%)		
	Agree	Neutral	Disagree	Agree	Neutral	Disagree
Vaccinating my children against diseases that can be spread from person to person is important (N = 219)	114 (99.1)	0 (0)	1 (0.9)	104 (100.0)	0 (0)	0 (0)
It is important for children and adolescents to be vaccinated against HPV (*N* = 214)	109 (97.3)	1 (0.9)	2 (1.8)	98 (96.1)	1 (1.0)	3 (2.9)
The HPV vaccine is beneficial for girls between 9 and 13 years old (*N* = 217)	107 (93.9)	1 (0.9)	6 (5.3)	97 (94.2)	2 (1.9)	4 (3.9)
The HPV vaccine is safe (N = 215)	100 (90.1)	5 (4.5)	6 (5.4)	87 (83.7)	12 (11.5)	5 (4.8)
The HPV vaccine is effective in preventing cervical cancer (*N* = 207)	98 (90.7)	4 (3.7)	6 (5.6)	95 (96.0)	3 (3.0)	1 (1.0)
The HPV vaccine is beneficial for women over the age of 13 (*N* = 209)	90 (83.3)	1 (0.9)	17 (15.7)	84 (83.2)	5 (4.9)	12 (11.9)
Men should be vaccinated against HPV (*N* = 213)	85 (74.9)	3 (2.7)	24 (21.4)	85 (84.2)	3 (3.0)	13 (12.9)
The HPV vaccine is beneficial for men (*N* = 202)	66 (62.3)	3 (2.8)	37 (34.9)	62 (64.6)	6 (6.2)	28 (29.2)

### Knowledge

Approximately 82% of parents knew that HPV is transmitted through sexual contact, and the majority of parents (71.4%) knew that both males and females should be vaccinated against HPV. Approximately 66% of parents could correctly identify the 2016 eligibility criteria for vaccination through SUS. Knowledge that women can be vaccinated up to age 26 was low, with only 47% of parents correctly responding to this item. There were no statistically significant knowledge differences between the two parent groups (*p* > 0.05) (Table 4).

**Table 4:** Knowledge about HPV, HPV vaccine, transmission, and Brazil’s eligible population for vaccination among parents with children between the ages of 9 and 17 in São Paulo, Brazil, 2015

	Overall N (%)	HPV vaccine eligible N (%)	HPV vaccine ineligible N (%)	*X* ^*2*^ *p*-value
HPV is transmitted through sexual contact (N = 207)				0.6320
True^a^	175 (82.2)	90 (81.1)	85 (83.3)	
False	23 (10.8)	14 (12.6)	9 (8.8)	
Don’t know	15 (7.0)	7 (6.3)	8 (7.8)	
Recommended that both males and females be vaccinated against HPV (*N* = 207)				0.2095
True^a^	152 (71.4)	75 (66.4)	77 (77.0)	
False	47 (22.1)	30 (26.5)	17 (17.0)	
Don’t know	14 (6.6)	8 (7.1)	6 (6.0)	
Currently eligible in Brazil to receive the HPV vaccine (*N* = 211)				0.5576
Only girls, 9–13^a^	141 (65.6)	73 (63.5)	68 (68.0)	
Only girls, 9–26	7 (3.3)	5 (4.3)	2 (2.0)	
Girls and boys 9–13	27 (12.6)	14 (12.2)	13 (13.0)	
Girls and boys, 9–26	30 (14.0)	19 16.5)	11 (11.0)	
Don’t know	10 (4.7)	4 (3.5)	6 (6.0)	
Girls can be vaccinated against HPV up to age 26 (N = 207)				0.6850
True^a^	100 (47.0)	50 (44.2)	50 (50.0)	
False	71 (33.3)	39 (34.5)	32 (32.0)	
Don’t know	42 (19.7)	24 (21.2)	18 (18.0)	

^a^ Indicates correct answer

## Discussion

Comparing the two parent groups in our study provides us with the opportunity to assess parental attitudes among those whose children are currently eligible for HPV vaccination with parents whose children are not. The exclusion of males poses a potentially discriminatory violation of the right to health underscoring the importance of the human rights principles of non-discrimination and participation in health policy making. Nearly universally, parents demonstrated support for the HPV vaccine outside of the current criteria. Almost 90% of parents expressed that they would have all of their children vaccinated against HPV, regardless of gender and up to age 26, if the vaccine was available through SUS. These findings are similar to the results of a qualitative study of Brazilian parents in 2014 [20]. Our data also elucidates two related challenges – financial and information accessibility within the context of widespread support for the expansion of HPV vaccine eligibility.

Financial accessibility – a key component of the right to health -- is an important factor; 94 % of parents expressed a desire to get their child vaccinated against HPV, if the vaccine were financially accessible. For those not currently eligible under the existing policy, HPV vaccination is likely out of their financial reach. With a cost of US$135 per HPV vaccine dose [21], it is not feasible to expect parents in a population with average monthly income of US$258 to pay over half their monthly income for one HPV vaccine dose (two to three doses are necessary for complete protection). These results highlight that the main barrier to HPV vaccination in Brazil is cost – both for the state government and individuals.

As Brazil moves towards its own production of the HPV vaccine, expansion of the HPV vaccine to other populations where the vaccine has proven efficacious must be considered [12]. Currently, eligibility for the SUS provided vaccine is determined by gender and age – potentially discriminatory determinants – creating a missed opportunity for the prevention of HPV infection and related cancers, in both males and females [15, 22]. In 2006, SUS paid over USD $538 million for screening, diagnosis and treatment of precancerous lesions, and the surgical and clinical treatment of cervical cancer [23]. Considering that approximately 50% of males in Brazil between the ages of 18 and 70 have some form of the virus, vaccination for males is imperative for the well-being of both genders [12]. Yet the financial accessibility concerns raised by our participants must not be forgotten. If the proposed social spending freeze amendment recently announced by the Brazilian government is adopted, it will disproportionately affect and harm the poor population by reducing expenditure on health care and other social programs [24]. Any reduction in the free provision of the HPV vaccine would undermine Brazil’s human rights commitments even more than the exclusion of males and females over 13 from universal HPV vaccination [5].

While cost was the biggest accessibility barrier to HPV vaccine uptake in our population of SUS users, there was no significant difference in knowledge of HPV transmission and vaccination between parent groups. Health knowledge, or information accessibility, while not always linked to vaccination uptake, is also a key component of the right to health [3, 20]. SUS, as the universal health system responsible for fulfillment of the right to health in Brazil, includes education to inform health decision-making [3, 12]. These results show that the low-income populations are receiving and synthesizing the information being provided to them and are able to make informed decisions about the HPV vaccine. Informed populations may be more likely to participate in public health programming and make demands related to the right to health. The fact that knowledge and attitudes were consistent across groups highlights the importance of addressing population attitudes and concerns about excluded populations, as both parent groups want the best possible health outcomes for their children. Both groups are also equally entitled to participate in health policy and program decision-making and these concerns should be considered in that context. Taken together these data are instructive for policy makers as they consider the perspectives of community members. Governments may consider the commission of population based attitudinal studies for generalizable and participatory inputs on health policies, programs and strategies. These data may be considered alongside cost-effectiveness and competing health priorities.

In addition to preventing the spread of HPV to female partners, vaccination against HPV can prevent negative sexual health outcomes for males [15]. Gender-neutral HPV vaccination is being called for globally as the prevalence of HPV-associated cancers continues to rise. Most recently, a Lancet study detailed the risk of excluding males from HPV immunization programs, and calling for inclusive HPV vaccination for all African nations [25]. As the first Latin American country to include male HPV vaccination in their national immunization program, Brazil is rightfully exemplifying how and gender must be considered as a part of fulfilling the right to health [26]. Although far from perfect, the Brazilian model shows how a rights-based approach to universal health care can improve population health, and simultaneously decrease state financial burden. The plan to remove age- and gender-based exclusions on the HPV vaccine reiterates Brazil’s commitment to the principle of non-discrimination; however, if the proposed amendment to freeze healthcare spending for 20 years is approved [24], Brazil may undo its hard-earned achievements towards equity and fulfillment of the right to health. In early 2017 the Brazilian Ministry of Health announced that HPV vaccination eligibility would be extended to males aged 12–13, and would gradually increase to include males aged 9 to 13 by 2020; HIV-positive males aged 9 to 26 and 14-year-old females who have not yet been vaccinated will also be eligible to receive vaccination through SUS [27]. This decision addresses at least in part some concerns relating to age- and gender-based discrimination. However, the 2018 election of a more conservative President may stall or undermine these efforts [28].

Emerging health challenges, such as the spread of Zika virus and its concurrent vaccine development, raise related questions about targeted eligibility (i.e. on the basis of gender) for vaccinations [29]. Like HPV, the Zika virus may be transmitted sexually and the health impacts of any potential gender-based exclusions to vaccination must be considered. While Zika infection is most dangerous to the fetuses of pregnant woman, making them a priority for vaccination, infected males may spread the virus through semen even after pregnancy. This highlights the potential for future research regarding perception of inequities in access; in the case of HPV, universal vaccination to all individuals for whom vaccine efficacy has been scientifically demonstrated has considerable benefits [12, 25, 30]. Moreover, as the research in the field is constantly evolving – in relation to vaccine efficacy and guidelines – governments should be prepared to continually evaluate current standards and be responsive as these recommendations evolve and expand over time. In the case of HPV, the recent FDA licensure of Gardasil 9 (HPV 9-valent vaccine) for use among men and women aged 27 to 45 offers a new and unique opportunity for protection in previously excluded populations, and against additional strains that may be acquired even after the onset of sexual activity [31].

Brazil’s HPV vaccination campaign has already been successful and influential. Prior to 2014, the prevalence of HPV infection was between 24.8 and 35.0% [32]. As of 2016, over 13 million Brazilian females are protected against the four types of HPV responsible for 90% of genital warts and 70% of cervical cancers [11]. In the municipality of Mauá, 85% of the eligible population received the first and second dose of the HPV vaccine in 2014 [11]. Vaccination rates have remained high in these communities, and if expanded to the excluded populations, population-level protection would be expected to increase. Such protections benefit individual and population health, as well as reducing expenditures by the public sector on cervical cancer screening and treatment. The adage, “an ounce of prevention is worth a pound of cure” has never been truer.

As with all studies, there are certain limitations to our data. Based on participant attitudes, it is evident that there is strong support for the expansion of HPV vaccination for excluded populations, and is not based on currently eligibility, or differential knowledge and attitudes about the vaccine. These data may be limited by social desirability bias. While most bias of this sort is related to self-reported behavior, it is possible that our participants assumed that the researchers would view expansion of HPV vaccine eligibility as favorable and thus responded accordingly. Moreover, our study shows only preliminary results on this topic, and due to the sample size and its cross-sectional nature, a larger, more representative study is required to generalize the findings to the entire country. It is important to note that the field has been widely researched and opposing studies exist both in support of gender-neutral vaccination, as well as, in support of vaccinating adolescent girls only and relying on herd immunity [33, 34]. There is no denying that men benefit from high HPV vaccination rates in women; however, in countries where vaccine uptake rates are low, selective immunization is unlikely to reach the level of herd immunity in which men will be protected as effectively as if they had been vaccinated [35]. With most HPV guidelines still exclusively focused on adolescent females, and only 35% of countries including the vaccine as part of national immunization programming, more research about the expansion of HPV vaccination among males is still needed [13, 25, 36].

## Conclusions

Since implementing SUS in 1988, Brazil has remained proactive in prioritizing health care and diminishing excessive costs for the individual, despite limited financial resources. To achieve its mandate for universal health access, SUS programs must be realized across age, gender, and income level [1]. As evidenced by this study, Brazilian parents are expressing great support for universal HPV vaccination; this should be addressed on the basis of the human rights principles of non-discrimination, participation, information and financial accessibility, as well as scientific efficacy. Such lessons are true not only for existing health threats such as HPV-related cancers, but emerging issues such as Zika virus [29].

Vaccination is a main component of preventive care, and based on multiple cost-effective studies, HPV vaccination is favorable [23, 37]. In time, implementation of the HPV vaccine into the PNI system will result in a significant reduction of cervical cancer incidence and mortality in Brazil, while decreasing future treatment costs for the government [23, 37]. The implications for universal HPV vaccination are incredible, and Brazil will see a decrease in genital warts, as well as mouth, throat, penile, and anal cancers in the male population in the decades to come, consequently decreasing healthcare spending in the process [26]. Additionally, the vaccination of males will aid in the prevention of HPV spread to unvaccinated females, reducing the incidence of cervical and vulvar cancer. In August 2017, the Brazilian government announced that it would begin expanding HPV vaccination to include males aged 11–15 and HIV positive persons up to age 26 making it the first country in South America to do so [27, 38]. Moving forward, careful consideration of age- and gender-based eligibility for vaccination, informed decision making and the importance of community participation in health policy development and implementation may be considered in Brazil and beyond.

## Abbreviations

FMABC: ABC School of Medicine *(Faculdade de Medicina do ABC)*
HPV: Human papillomavirus
OOP: Out of pocket
PNI: National Immunization Program (*Programa Nacional de Imunização)*
SUS: Unified Health System (*Sistema Único de Saúde*)
UHC: Universal Health Coverage
WHO: World Health Organization
